# Glucose levels during gestational diabetes pregnancy and the risk of developing postpartum diabetes or prediabetes

**DOI:** 10.1186/s12884-021-04352-w

**Published:** 2022-01-08

**Authors:** Chadakarn Phaloprakarn, Siriwan Tangjitgamol

**Affiliations:** Department of Obstetrics and Gynecology, Faculty of Medicine Vajira Hospital, Navamindradhiraj University, 681 Samsen Road, Dusit District, Bangkok, 10300 Thailand

**Keywords:** Gestational diabetes mellitus, Glucose level, Glycemic control, Postpartum prediabetes, Postpartum type 2 diabetes mellitus

## Abstract

**Background:**

Blood glucose levels during pregnancy may reflect the severity of insulin secretory defects and/or insulin resistance during gestational diabetes mellitus (GDM) pregnancy. We hypothesized that suboptimal glycemic control in women with GDM could increase the risk of postpartum type 2 diabetes mellitus (T2DM) or prediabetes. Our objective was to evaluate the impact of plasma glucose levels throughout GDM pregnancy on the risk of postpartum T2DM or prediabetes.

**Methods:**

The medical records of 706 women with GDM who underwent a postpartum 75-g, 2-hour oral glucose tolerance test at our institution between January 2011 and December 2018 were reviewed. These women were classified into 2 groups according to glycemic control during pregnancy: ≤ 1 occasion of either fasting glucose ≥ 95 mg/dL or 2-hour postprandial glucose ≥ 120 mg/dL was defined as optimal glycemic control or else was classified as suboptimal glycemic control. Rates of postpartum T2DM and prediabetes were compared between women with optimal (n = 505) and suboptimal (n = 201) glycemic control.

**Results:**

The rates of postpartum T2DM and prediabetes were significantly higher in the suboptimal glycemic control group than in the optimal glycemic control group: 22.4% vs. 3.0%, *P* < 0.001 for T2DM and 45.3% vs. 23.5%, *P* < 0.001 for prediabetes**.** In a multivariate analysis, suboptimal glucose control during pregnancy was an independent risk factor for developing either postpartum T2DM or prediabetes. The adjusted odds ratios were 8.4 (95% confidence interval, 3.5–20.3) for T2DM and 3.9 (95% confidence interval, 2.5–6.1) for prediabetes.

**Conclusion:**

Our findings suggest that blood glucose levels during GDM pregnancy have an impact on the risk of postpartum T2DM and prediabetes.

## Background

Gestational diabetes mellitus (GDM) is a common metabolic disorder in pregnancy that affects 20% to 25% of Southeast Asian pregnant women [1]. This metabolic derangement is characterized by insufficient insulin secretion from pancreatic β-cells to compensate for pregnancy-induced physiologic insulin resistance [2]. The coexistence of insulin secretory defects and insulin resistance can raise maternal blood glucose levels, resulting in adverse pregnancy outcomes [3, 4]. These coexisting disorders are also well recognized as important factors in the pathogenesis of prediabetes and type 2 diabetes mellitus (T2DM) [5, 6].

Good control of plasma glucose levels throughout gestation among pregnant women with GDM is generally recommended to minimize adverse pregnancy outcomes. Earlier studies have found increased risks of macrosomia and cesarean delivery among women with suboptimal or uncontrolled blood glucose levels compared to women with optimal glycemic control [7, 8]. Aside from pregnancy outcomes, an increased risk of postpartum glucose intolerance or T2DM in women with GDM who had poor glycemic control during pregnancy has been reported [9, 10]. However, the definition of ‘good or optimal’ vs. ‘poor or suboptimal’ glucose levels (using the 2-hour postprandial vs. mean daily glucose level) and the time points for the diagnosis or development of T2DM (weeks vs. years postpartum) are inconsistent among previous studies [9, 10].

Given that the levels of blood glucose during pregnancy may reflect the severity of insulin secretory defects and/or insulin resistance during GDM pregnancy [11], we hypothesized that suboptimal glycemic control in women with GDM would impart an increased risk of postpartum T2DM or prediabetes. The aim of this study was to determine the impact of glucose levels during GDM pregnancy on the risk of developing T2DM or prediabetes at 6 weeks postpartum. The criteria for optimal and suboptimal glycemic control were based on the standard recommendations of the American Diabetes Association (ADA) and the American College of Obstetricians and Gynecologists (ACOG) that the fasting plasma glucose (FPG) in GDM pregnancy should be maintained below 95 mg/dL and the 1-hour or 2-hour postprandial glucose below 140 mg/dL or 120 mg/dL, respectively [12, 13].

## Methods

### Participants

This retrospective study was performed at the Faculty of Medicine Vajira Hospital, which is an 800-bed tertiary care and referral hospital located in Bangkok, Thailand. Medical records of women with a diagnosis of GDM who delivered and returned to our institution between January 2011 and December 2018 for a 75-g, 2-hour oral glucose tolerance test (OGTT) at 6 weeks postpartum were reviewed. The inclusion criterion was women who had at least four occasions of blood glucose measurements during pregnancy. Women with a diagnosis of pregestational diabetes and those without available data on fasting and/or postprandial plasma glucose levels during pregnancy were excluded from the study.

### Blood glucose testing and diagnosis

As a standard practice in our institution, all pregnant women without risk factors for GDM underwent a glucose challenge test (GCT) at 24–28 weeks of gestation. On the other hand, women with risk factors received a screening test at the initial visit and, if negative, were rescreened at 28–32 weeks of gestation. Individuals with a GCT result of 140 mg/dL or higher would be scheduled for a diagnostic 100-g OGTT. The diagnosis of GDM was based on the Carpenter and Coustan criteria [14].

The management of women with GDM included dietary and lifestyle modifications as initial treatment. These women were evaluated for their levels of glycemic control throughout gestation by the measurement of fasting and 2-hour postprandial plasma glucose levels every 2–4 weeks. Insulin therapy, as determined and prescribed by an endocrinologist, was generally initiated when the FPG was ≥ 95 mg/dL and/or 2-hour postprandial glucose was ≥ 120 mg/dL. Factors that were also involved in the decision to initiate insulin treatment were the compliance of pregnant women with insulin injections, gestational age at which abnormally high FPG or 2-hour postprandial glucose was detected, and type (fasting or postprandial glucose), magnitude and number of hyperglycemic episodes.

Optimal or suboptimal control of plasma glucose levels was defined according to the thresholds for fasting and 2-hour postprandial glucose recommended by the ADA and ACOG [12, 13]. Optimal glycemic control was defined as no more than one occasion of either an FPG level of at least 95 mg/dL or a 2-hour postprandial glucose level of at least 120 mg/dL. Suboptimal glycemic control was defined as two or more occasions of an FPG level of at least 95 mg/dL and/or a 2-hour postprandial glucose level of at least 120 mg/dL.

After delivery, all women with a diagnosis of GDM were scheduled for a 75-g, 2-hour OGTT at 6 weeks postpartum.

### Data collection and study outcome

The data were extracted from medical charts and the hospital’s electronic database. These included age, parity, prepregnancy body mass index (BMI), history of T2DM in any first-degree relatives, GCT and 100-g OGTT values, gestational age at GDM diagnosis, insulin use (type and dose), fasting and 2-hour postprandial glucose levels throughout pregnancy, neonatal birth weight, and levels of postpartum 75-g, 2-hour OGTT, which included an FPG and a plasma glucose at 2 hours after the 75-g glucose load. Data on individual glucose levels throughout pregnancy were collected from regular blood glucose monitoring during antenatal visits.

The primary study outcome was the development of T2DM or prediabetes at 6 weeks postpartum. The diagnosis of T2DM was a level of FPG of at least 126 mg/dL or a plasma glucose level of at least 200 mg/dL at 2 hours after a 75-g OGTT [13]. An FPG level of 100–125 mg/dL or a 2-hour glucose level of 140–199 mg/dL was referred to as impaired fasting glucose or impaired glucose tolerance, respectively. The diagnosis of prediabetes included either impaired fasting glucose or impaired glucose tolerance or both [13].

### Statistical analysis

Statistical analysis was performed using IBM SPSS Statistics for Windows, Version 22.0 (IBM Corporation, Armonk, NY, USA). Categorical variables are described as numbers and percentages and were compared by the chi-square test or Fisher’s exact test as appropriate. Continuous variables are reported as the median and interquartile range and were compared with the Mann-Whitney U test. The odds ratios (ORs) with 95% confidence intervals (CIs) of T2DM and prediabetes of suboptimal glycemic control were analyzed by multivariate logistic regression and adjusted for potential confounders. The predictive performance of suboptimal glycemic control during pregnancy for postpartum T2DM or prediabetes was determined by the receiver operating characteristic (ROC) curve. A value of *P* < 0.05 was considered statistically significant.

### Ethical approval

This study was approved by the Vajira Institution Review Board (certificate of approval no.138/2561) and was performed in compliance with the Declaration of Helsinki.

## Results

### Characteristics of the study population

A total of 706 women with GDM who had available data on plasma glucose levels during pregnancy and measurements of postpartum FPG and 75-g, 2-hour OGTT were included for analysis. Of these, 505 (71.5%) women had optimal blood glucose control, and 201 (28.5%) had suboptimal glucose control during pregnancy. The baseline characteristics of women with optimal and suboptimal glycemic control are presented in Table 1. Rates of multiparity, overweight or obese BMI (≥ 25 kg/m^2^), history of T2DM in any first-degree relatives, early-onset GDM (< 24 weeks), and insulin use were significantly higher among women with suboptimal glycemic control than those with optimal glycemic control. The women with suboptimal glycemic control also had significantly higher median prepregnancy BMI, GCT, fasting, 1-hour and 2-hour OGTT values, units of insulin used, and neonatal birth weight but a significantly lower gestational age at GDM diagnosis.

**Table 1:** Characteristics of women with optimal and suboptimal glycemic control

Characteristic	Optimal glycemic control	Suboptimal glycemic control	*P*
	(*n* = 505)	(*n* = 201)	
Age (years)	32.0 (28.0–36.0)	33.0 (28.0–37.0)	0.514
≥ 35 years old	190 (37.6)	82 (40.8)	0.434
Multiparity	294 (58.2)	149 (74.1)	< 0.001
Prepregnancy BMI (kg/m^2^)	22.6 (20.4–25.7)	26.3 (23.0–30.7)	< 0.001
Overweight or obese BMI (≥ 25 kg/m^2^)	146 (28.9)	119 (59.2)	< 0.001
History of T2DM in any first-degree relatives	135 (26.7)	69 (34.3)	0.045
GCT value (mg/dL)	164.0 (152.0–182.0)	177.0 (156.5–210.0)	< 0.001
100-g OGTT (mg/dL)			
Fasting value	82.0 (75.0–88.8)	96.0 (88.0–106.0)	< 0.001
1-hour value	191.0 (180.0–204.0)	207.0 (191.0–232.0)	< 0.001
2-hour value	173.0 (160.0–188.0)	183.0 (167.0–213.0)	< 0.001
3-hour value	146.5 (127.0–160.0)	148.0 (125.0–174.0)	0.134
Gestational age at GDM diagnosis (weeks)	29.0 (17.0–31.0)	18.5 (10.0–29.0)	< 0.001
Early-onset GDM (< 24 weeks)	156 (30.9)	121 (60.2)	< 0.001
Insulin use	12 (2.4)	82 (40.8)	< 0.001
Type of insulin			
Rapid-acting insulin	1 (0.2)	10 (5.0)	< 0.001
Short-acting insulin	0 (0)	22 (10.9)	< 0.001
Intermediate-acting insulin	4 (0.8)	20 (10.0)	< 0.001
Premixed insulin	8 (1.6)	51 (25.4)	< 0.001
Units of insulin used in pregnancy	333 (130–411)	946 (303–1758)	0.006
Average plasma glucose during pregnancy (mg/dL)			
Fasting value	78.9 (74.0–84.0)	92.5 (84.6–100.9)	< 0.001
2-hour postprandial value	98.0 (89.5–106.0)	119.5 (111.4–132.5)	< 0.001
Neonatal birth weight (g)	3220 (2905–3549)	3516 (3039–3840)	< 0.001

Data are expressed as the median (IQR) or n (%).

*BMI* Body mass index; *GCT* Glucose challenge test; GDM: Gestational diabetes mellitus; *IQR* Interquartile range; *OGTT* Oral glucose tolerance test; *T2DM* Type 2 diabetes mellitus

### Rates of postpartum T2DM and prediabetes

The rates of postpartum T2DM and prediabetes in women who had suboptimal blood glucose control during pregnancy were significantly higher than those in women with optimal blood glucose control: 22.4% vs. 3.0% for T2DM and 45.3% vs. 23.5% for prediabetes, *P* < 0.001 for both (Table 2).

**Table 2:** Risks of postpartum type 2 diabetes mellitus or prediabetes in women with optimal and suboptimal glycemic control

Glucose control	Postpartum risk					
	Either T2DM or prediabetes (*n* = 270)		T2DM (*n* = 60)		Prediabetes (*n* = 210)	
	*n* (%)	Adjusted OR^a^	*n* (%)	Adjusted OR^a^	*n* (%)	Adjusted OR^a^
		(95% CI)		(95% CI)		(95% CI)
Optimal glycemic control (*n* = 505)	134/505 (26.5)	1.0 (reference)	15/505 (3.0)	1.0 (reference)	119/505 (23.5)	1.0 (reference)
Suboptimal glycemic control (*n* = 201)	136/201 (67.7)	4.4 (2.9–6.8)	45/201 (22.4)	8.4 (3.5–20.3)	91/201 (45.3)	3.9 (2.5–6.1)
Pattern of suboptimal glycemic control						
Either fasting or postprandial glucose (*n* = 66)	39/66 (59.1)	3.7 (2.1–6.5)	4/66 (6.1)	2.6 (0.5–13.6)	35/66 (53.0)	3.9 (2.2–7.0)
Both fasting and postprandial glucose (*n* = 135)	97/135 (71.9)	5.3 (3.1–9.1)	41/135 (30.4)	15.0 (5.4–41.7)	56/135 (41.5)	4.0 (2.3–7.1)

*BMI* Body mass index; *CI* Confidence interval; *GCT* Glucose challenge test; *GDM* Gestational diabetes mellitus; *OGTT* Oral glucose tolerance test; *OR* Odds ratio; *T2DM* Type 2 diabetes mellitus

^a^Adjusted for parity, prepregnancy BMI, history of T2DM in any first-degree relatives, early-onset GDM, GCT and 100-g OGTT glucose values.

After adjustment for potential confounding factors (parity, prepregnancy BMI, history of T2DM in any first-degree relatives, early-onset GDM, GCT and 100-g OGTT glucose values), suboptimal glucose control during pregnancy was an independent risk factor for developing either postpartum T2DM or prediabetes. The adjusted ORs were 8.4 (95% CI, 3.5–20.3) for T2DM and 3.9 (95% CI, 2.5–6.1) for prediabetes. Women with suboptimal control of either fasting or postprandial glucose during pregnancy had a lower OR for developing either T2DM or prediabetes than women with suboptimal control of both fasting and postprandial glucose levels: 3.7 (95% CI, 2.1–6.5) vs. 5.3 (95% CI, 3.1–9.1). Other independent risk factors for developing either postpartum T2DM or prediabetes were early-onset GDM and abnormal 100-g OGTT results, particularly abnormal fasting and 3-hour glucose values. The adjusted ORs were 1.5 (95% CI, 1.1–2.2), 1.6 (95% CI, 1.1–2.5) and 2.2 (95% CI, 1.5–3.2), respectively.

### Comparison of different glucose thresholds for suboptimal glycemic control

**Fig. 1:**
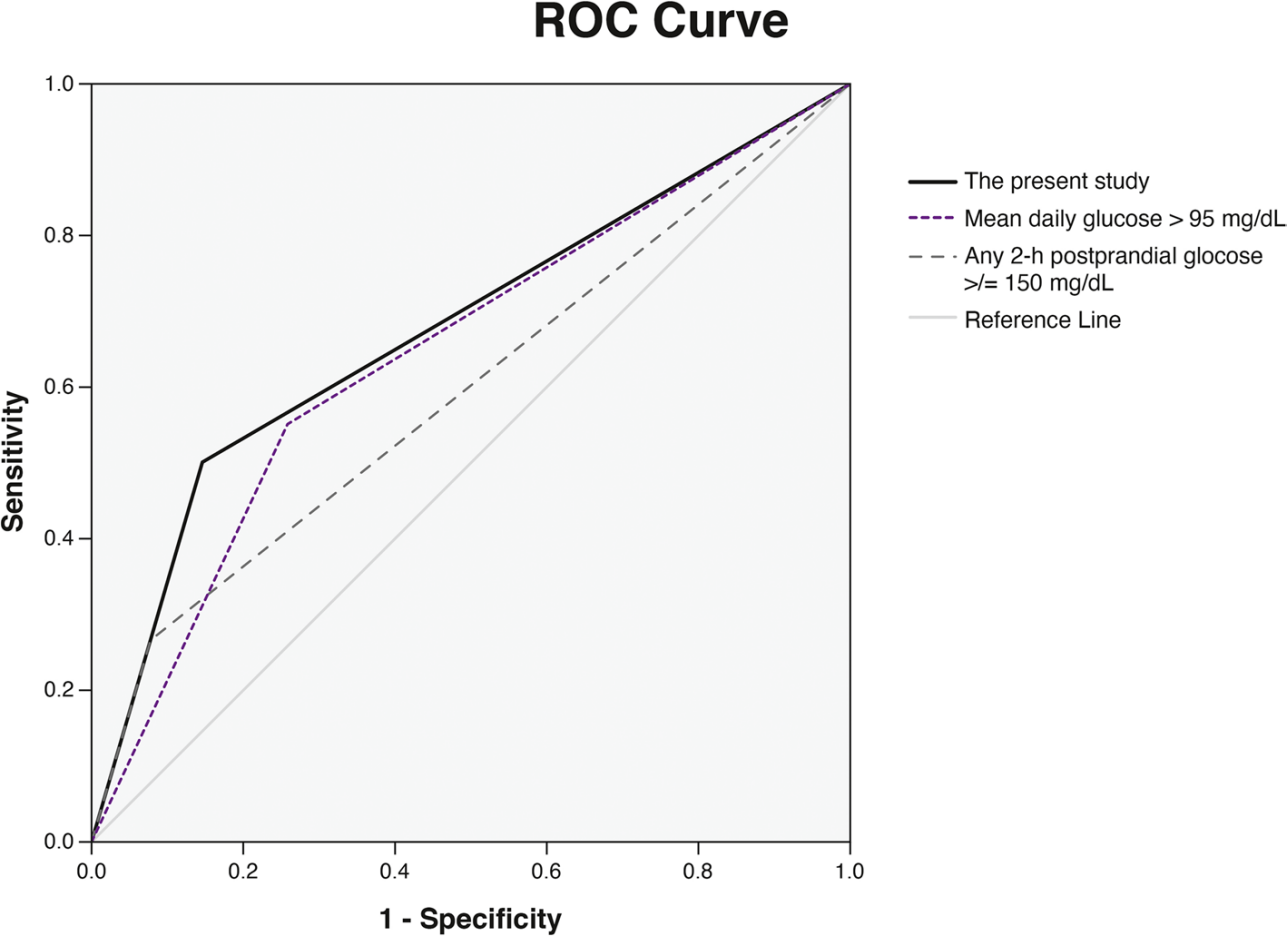
Receiver operating characteristic curves of the three criteria used to define suboptimal glycemic control for the prediction of postpartum type 2 diabetes mellitus or prediabetes

We also compared the predictive performances of postpartum T2DM or prediabetes of the three different glucose thresholds for suboptimal glycemic control: the thresholds being used in the present study (recommended by the ADA and ACOG), the threshold using any 2-hour postprandial glucose level of 150 mg/dL or higher [9] and the threshold using a mean daily glucose of more than 95 mg/dL [10]. The glucose thresholds recommended by the ADA and ACOG yielded the best predictive performance with an area under the ROC curve of 0.677 (95% CI 0.635–0.720), followed by the threshold using a mean daily glucose of more than 95 mg/dL [10] and the threshold using any 2-hour postprandial glucose level of 150 mg/dL or higher [9] (Fig. 1). The sensitivities, specificities, positive predictive values, negative predictive values, and area under the ROC curves of the three predictive criteria are presented in Table 3.

**Table 3:** Predictive performance for postpartum type 2 diabetes mellitus or prediabetes according to the three criteria for defining suboptimal glycemic control

Criteria	Sensitivity (%)	Specificity (%)	PPV (%)	NPV (%)	AUC
	(95% CI)	(95% CI)	(95% CI)	(95% CI)	(95% CI)
The present study^a^	50.4 (44.3–56.5)	85.1 (81.4–88.3)	67.7 (61.9–73.0)	73.5 (68.3–75.1)	0.677 (0.635–0.720)
Any 2-hour postprandial glucose ≥ 150 mg/dL	26.7 (21.5–32.4)	92.2 (89.3–94.5)	67.9 (59.2–75.6)	67.0 (65.3–68.7)	0.594 (0.550–0.639)
Mean daily glucose > 95 mg/dL	55.2 (49.0–61.2)	74.1 (69.7–78.1)	56.9 (52.1–61.5)	72.8 (69.8–75.5)	0.646 (0.604–0.689)

*AUC* Area under the receiver operating characteristic curve; *CI* Confidence interval; *NPV* Negative predictive value; *PPV* Positive predictive value

^a^The criteria for defining suboptimal glycemic control used in the present study were ≥ 2 occasions of fasting glucose ≥ 95 mg/dL and/or 2-hour postprandial glucose ≥ 120 mg/dL.

## Discussion

The findings of this study indicated that blood glucose levels throughout pregnancy of women with GDM were directly related to the risk of developing T2DM or prediabetes at 6 weeks postpartum. This association was independent of the glucose levels measured at the time of GDM diagnosis, as assessed by the 100-g OGTT values. Our observation suggests that not merely the presence of GDM, which is a well-known risk factor for T2DM [15], but also glycemic control plays an important role in modifying the risk of postpartum T2DM as well as prediabetes. Pregnant women with GDM should be educated about the benefits and targets of optimal glycemic control to decrease both intra- and postpartum adverse effects. Regular monitoring of plasma glucose levels and timely initiation of insulin therapy should be considered to maintain adequate glycemic control throughout pregnancy.

Furthermore, we found a direct association between the degree of suboptimal glycemic control, as characterized by the pattern of elevated fasting and postprandial glucose levels, and the development of T2DM or prediabetes postpartum. Risks of postpartum T2DM or prediabetes were increased by 3.7-fold among women with suboptimal control of either fasting or postprandial glucose and 5.3-fold among women with suboptimal control of both fasting and postprandial glucose compared to women with optimal glycemic control.

Two hypotheses have been proposed to explain the association between suboptimal glycemic control during GDM pregnancy and the development of postpartum T2DM or prediabetes. First, this may be related to the severity of chronic β-cell dysfunction and insulin resistance that manifests as hyperglycemia in pregnancy [11, 16, 17] and translates into a continuum of dysglycemia (prediabetes and T2DM) in the postpartum period or later in life [16, 17]. Saisho et al. [11], who used the disposition index to evaluate β-cell dysfunction, found an association of the level of β-cell dysfunction with the severity of glucose intolerance (assessed by the fasting and mean daily blood glucose levels) during pregnancy among Japanese women with GDM. Other authors also observed a correlation between the degree of glucose intolerance in pregnancy and the magnitudes of β-cell dysfunction and insulin resistance in Western pregnant women [16, 17]. Given that insulin secretory defects and insulin resistance are well recognized as precursors of T2DM and prediabetes [5, 6], the risk of developing either type of glucose intolerance postpartum was therefore higher in women with a more severe degree of suboptimal glycemic control during pregnancy than in those with a lesser extent of suboptimal glycemic control.

The second hypothesis is that chronic exposure of pancreatic islets to elevated glucose levels during pregnancy exerts toxicity on β-cells by oxidative stress, leading to more β-cell dysfunction and cell death and consequent prediabetes or T2DM after pregnancy [18, 19]. These cascade events were demonstrated in several studies that found a reduction in β-cell volume among patients with T2DM compared to nondiabetic subjects [20–23]. Nevertheless, such prior studies were limited by being a cross-sectional research design and not being conducted *in vivo*. Further longitudinal studies are therefore needed to compare the changes in β-cell mass over time from antepartum to postpartum among women with GDM who develop and do not develop postpartum T2DM or prediabetes.

Given that there have been no recommendations from the expert panels regarding plasma glucose thresholds during pregnancy that are related to a reduced risk of postpartum T2DM or prediabetes, we then adopted the fasting and postprandial glucose targets, as recommended by the ADA and ACOG to reduce the risk of macrosomia [12, 13], to classify the plasma glucose levels of women in this study as indicating optimal or suboptimal glycemic control. Previous studies have reported that intermittent high glucose levels rather than persistent hyperglycemia stimulate reactive oxygen species overproduction, β-cell apoptosis and dysfunction [24–26]. Hence, we focused on high blood glucose levels at different time points in preference over high mean blood glucose levels. In our study, optimal glycemic control was defined as no more than one occasion of elevated FPG or 2-hour postprandial glucose. Several studies used less than three occasions of elevated glucose values and some clinics use less than 50% of elevated values out of all determined values to define optimal control [9, 27]. We selected this strict criterion aiming to alert the woman and her obstetrician to be cautious about the condition, so diet or behavioral modifications would be more focused.

With a fasting glucose level of 95 mg/dL or higher and/or 2-hour postprandial glucose of 120 mg/dL or higher on at least two occasions, the predictive performance of these glucose thresholds for postpartum T2DM or prediabetes yielded moderate discriminatory power (an area under the ROC curve of 0.677). Aside from these glucose thresholds, two other studies that used different glucose thresholds to define suboptimal glycemic control during pregnancy also reported an increased risk of postpartum glucose intolerance or future T2DM among women with suboptimal glycemic control [9, 10]. When a comparison among the three glucose thresholds was made, we found that the thresholds used in our study yielded the best predictive performance for T2DM or prediabetes at 6 weeks postpartum. Our findings suggested that the fasting and 2-hour postprandial glucose targets recommended by the ADA and ACOG to reduce the risk of macrosomia could also lessen the risk of developing postpartum T2DM or prediabetes.

Aside from T2DM, we also paid attention to the development of prediabetes because this metabolic state represents an intermediate hyperglycemic state for progression to T2DM [28]. As the lifetime risk of progressing from prediabetes to T2DM has been reported to be high at 74% [29], a reduced risk of postpartum prediabetes should be as important as that of T2DM. This study found that both spectra of glucose intolerance were significantly decreased with optimal glycemic control.

Notably, the present study focused specifically on the development of dysglycemia at 6 weeks postpartum, which is the usual time for a postpartum checkup. Screening for T2DM or prediabetes after delivery was performed in the same setting for convenience and early detection of any dysglycemic condition. Although data on the long-term impact of glycemic control during pregnancy on glucose intolerance are interesting, we were aware of other factors that may influence the risk of subsequent T2DM or prediabetes, such as diet and physical activity. A well-designed prospective study controlling all relevant factors with a long-term follow-up may be able to provide a definite answer regarding an association of glycemic control during pregnancy and the long-term risk of dysglycemia.

The strength of our study included having a large number of pregnant women with multiple fasting and postprandial blood glucose measurements throughout pregnancy. In addition, all blood glucose specimens collected in our institution were measured using the same automated glucose analyzer, which was calibrated regularly to obtain accurate results. Furthermore, the diagnoses of GDM and postpartum T2DM or prediabetes were made according to the standard guidelines. Nevertheless, there are a few limitations that one should bear in mind when applying our results in clinical practice. Although frequent blood glucose monitoring is an integral part of achieving glycemic targets, only blood glucose levels during antenatal visits were used in this study. This was because most women in our study had limited ability or resources to do frequent self-monitoring of their blood glucose levels. In this situation, a regular blood glucose measurement during antenatal care may be needed to define optimal or suboptimal glycemic control. Second, our study was limited by the use of Carpenter and Coustan criteria to diagnose GDM. We cannot confirm the validity of our findings in settings that use different approaches to GDM screening and diagnosis. Lastly, some may question the possibility of unrecognized pregestational diabetes among these women. It was less likely in our study because all of the study population had an FPG less than 126 mg/dL (before ingestion of the 100-g glucose load) and no one (among a few who had available hemoglobin A1c test) had an A1c of 6.5% or higher [30, 31].

## Conclusion

Our study demonstrated that glucose levels during GDM pregnancy have an impact on the risk of developing T2DM or prediabetes at 6 weeks postpartum. Optimal glycemic control throughout pregnancy is therefore necessary to reduce these risks. Aside from weight loss and insulin therapy that can temporarily improve β-cell function, future research is needed to search for interventions that can permanently arrest the progression of β-cell dysfunction in women with GDM.

## Data Availability

The datasets used and analyzed in this study are available from the corresponding author on reasonable request.

## Abbreviations

ACOG: American College of Obstetricians and Gynecologists
ADA: American Diabetes Association
BMI: Body mass index
CI: Confidence interval
FPG: Fasting plasma glucose
GCT: Glucose challenge test
GDM: Gestational diabetes mellitus
OGTT: Oral glucose tolerance test
OR: Odds ratio
ROC: Receiver operating characteristic
T2DM: Type 2 diabetes mellitus
